# Frugivore Traits Predict Plant–Frugivore Interactions Using Generalized Joint Attribute Modeling

**DOI:** 10.1002/ece3.70772

**Published:** 2025-01-16

**Authors:** Laurel R. Yohe, Leith B. Leiser‐Miller, Zofia A. Kaliszewska, Susan R. Whitehead, Sharlene E. Santana, Liliana M. Dávalos

**Affiliations:** ^1^ Department of Bioinformatics and Genomics University of North Carolina Charlotte Charlotte North Carolina USA; ^2^ North Carolina Research Campus Kannapolis North Carolina USA; ^3^ Department of Ecology & Evolution Stony Brook University Stony Brook New York USA; ^4^ Department of Earth and Planetary Sciences Yale University New Haven Connecticut USA; ^5^ Department of Biology University of Washington Seattle Washington USA; ^6^ Department of Biological Sciences Virginia Tech Blacksburg Virginia USA; ^7^ Burke Museum of Natural History and Culture University of Washington Seattle Washington USA; ^8^ Consortium for Inter‐Disciplinary Environmental Research, School of Marine and Atmospheric Sciences Stony Brook University Stony Brook New York USA

**Keywords:** bats, Bayesian hierarchical models, *Carollia*, dispersal syndrome, functional traits, mutualism, Phyllostomidae, *Piper*, seed dispersal, trophic interactions

## Abstract

Under an adaptive hypothesis, the reciprocal influence between mutualistic plants and frugivores is expected to result in suites of matching frugivore and plant traits that structure fruit consumption. Recent work has suggested fruit traits can represent adaptations to broad groups of functionally similar frugivores, but the role of frugivore traits and within‐species variation in structuring fruit consumption is less understood. To address these knowledge gaps, we assess the presence of reciprocal trait matching for the mutualistic ecological network comprising of *Carollia* bats that feed on and disperse *Piper* seeds. We used generalized joint attribute modeling (GJAM), a Bayesian modeling approach that simultaneously accounts for multiple sources of variance across trait types. In support of frugivore adaptation to their dietary composition and suggesting niche partitioning among *Carollia* bats, we find differential consumption of a suite of *Piper* species influenced by bat traits such as body size; however, the *Piper* morphological traits considered had no effect on bat consumption. Slow evolutionary rates, dispersal by other vertebrates, and unexamined fruit traits, such as *Piper* chemical bouquets, may explain the lack of association between bat *Piper* consumption and fruit morphological traits. We have identified a potential asymmetric influence of frugivore traits on plant–frugivore interactions, providing a template for future trait analyses of plant–animal networks. As intraspecific trait variation is rarely included in studies on trait matching, this paper contributes to closing that important knowledge gap.

## Graphical abstract

A *Carollia perspicillata* bat feeding on a fruit of Piper hispidum. Photo by Marco A. R. Mello (https://marcomellolab.wordpress.com), used with permission.

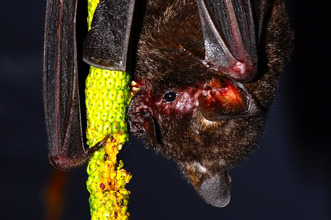

## 1 Introduction

A reciprocal influence between frugivores and fruit traits is often expected in ecological interactions comprised of seed dispersers and plant mutualists (Janson 1983). But frugivores may be generalists and their within‐population variation can obscure how organismal traits influence such interactions. Further, across rich ecological networks such as those in the Neotropics, an adaptive hypothesis for traits linking plant and animal species may be unwarranted. Instead, ecological fitting, whereby fruit–frugivore interactions emerge through the matching of ancestral traits to a new environment, could explain contemporary interactions without the need to invoke adaptation (Janzen 1985). There is strong support for animals shaping suites of fruit traits, *i.e.*, the dispersal syndrome hypothesis, in the form of fruit or seed size, hardness, color, and scent chemical profile matching of frugivore preferences (Valenta and Nevo 2020). Broad sensory, digestive, and excretory adaptations to frugivory are also well supported among vertebrate species (Herrera 1984; Schondube, Herrera‐M, and Martínez del Rio 2001; Saldaña‐Vázquez et al. 2013; Wang et al. 2020; Yohe et al. 2021). However, individual variation that may contribute to structuring mutualistic networks has often been ignored.

Testing for potential adaptive trait matching is further complicated by the multiple scales at which interactions and traits are measured. While the selective influence of frugivory on plants has been examined through seed dispersal and recruitment analyses (Norconk, Grafton, and Conklin‐Brittain 1998; Nathan and Muller‐Landau 2000; Howe and Miriti 2004), its effects on frugivore traits have been analyzed at scales that range from individuals to clades (Pratt and Stiles 1985; Stevenson, Quiñones, and Ahumada 2000; Burns 2004). Important gaps emerge from this variation in scales. Further, in contrast to plant–pollinator interactions, there are fewer studies of fruit–frugivore interactions, and many overlook within‐species variation. To date, research on the evolutionary consequences of fruit–frugivore interactions has primarily focused on traits, such as vertebrate color vision and fruit color indicating ripeness, that explain the foraging behavior of birds and diurnal mammals (Osorio et al. 2004; Schaefer, Schaefer, and Vorobyev 2007). In contrast, the influence of fruit traits on nocturnal frugivores (*e.g.*, bats) is largely unknown (Luft, Curio, and Tacud 2003; Hodgkison et al. 2013; but see Thies and Kalko 2004), even though bats constitute a large percentage of seed dispersers in tropical ecosystems (Fleming and Heithaus 1981; Muscarella and Fleming 2007; Fleming and John Kress 2011).

**FIGURE 1:**
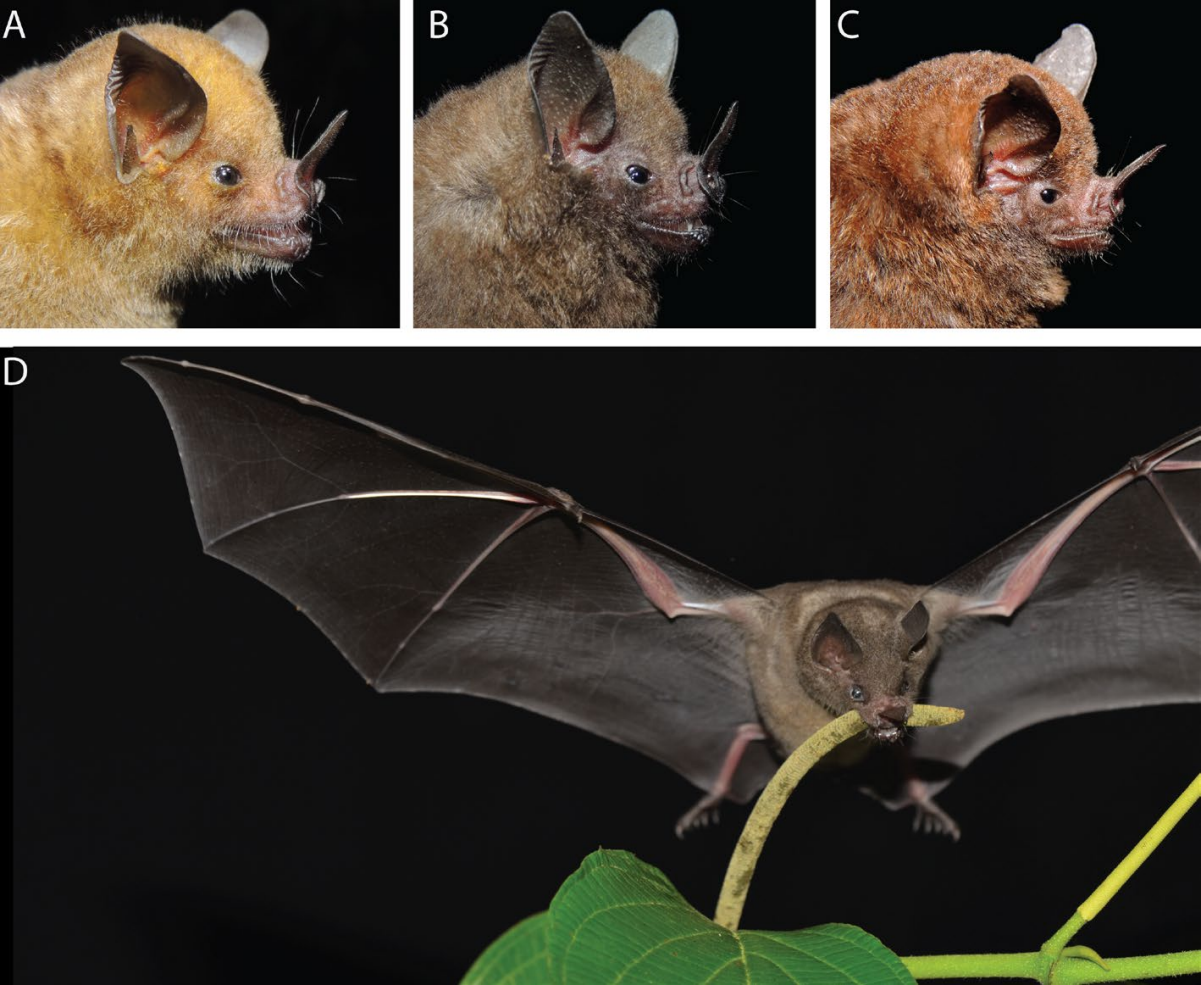
Headshots of the three sympatric short‐tailed fruit bats (*Carollia*) found in our study locality in Costa Rica: (A) *Carollia perspicillata* , (B) *C. sowelli* , and (C) *C. castanea* . (D) *C. perspicillata* feeding on Piper *sancti‐felicis*. Photo credit: David Villalobos Chaves (A–C) and Susan Whitehead (D).

We seek to better understand the extent to which animals have influenced trait evolution of their mutualistic plants and vice versa, leading to suites of matching traits that structure fruit–frugivore networks in diverse ecological communities. Here, we focus on neotropical *Piper* plants (Piperales: Piperaceae) and *Carollia* bats (Figure 1; Chiroptera: Phyllostomidae), a model mutualistic system whose ecology has been well documented. *Piper* are both diverse and abundant in tropical ecosystems worldwide (Gentry 1988) and provide a constant supply of ripe fruit throughout the year through continuous or staggered fruiting patterns among sympatric species (Thies and Kalko 2004). Many neotropical *Piper* species depend on *Carollia* for seed dispersal (Dyer and Palmer 2004), and *Piper* fruits dominate these bats' diets throughout the year and across their range (Fleming 1991). Because of the relative strength of this interaction compared to other fruit–frugivore interactions, many of which are highly diffuse in nature, this system is ideal for testing the potential for reciprocal adaptation and trait matching between fruits and frugivores.

In the *Piper*–*Carollia* interaction, the general mutualistic benefits of seed dispersal for *Piper* and nutritional rewards for *Carollia* have been well‐documented (Fleming 1988, 2004); however, the system presents a natural gradient of ecological interdependencies. At La Selva Biological Reserve in Costa Rica (10.431930, −84.004745), three species of co‐existing *Carollia* feed on at least a dozen *Piper* species, with *C. perspicillata* (Linneas, 1758; Gray 1838, 183; Cloutier and Thomas 1992) being the most generalist frugivore, *C. sowelli* (Baker, Hoffmann, and Solari 2002) being intermediate, and *C. castanea* (Allen 1890) being the most specialized on *Piper* (Fleming 1991). While behavioral studies of *Carollia* bats suggest adaptations to *Piper* scent cues (Thies, Kalko, and Schnitzler 1998; Leiser‐Miller et al. 2020), broad dietary overlap among the three bat species imply minimal specialization, leaving little room for differential frugivore adaptation (Maynard et al. 2019). Further, the mutualistic interactions did not necessarily evolve synchronously, leaving room to explore the variation in mechanisms structuring the interactions. The *Carollia* genus evolved less than 20 million years ago (Rojas, Warsi, and Dávalos 2016). Neotropical *Piper* emerged during the Oligo‐Miocene (Jaramillo et al. 2008), though the major diversification of *Piper* occurred as bat frugivores diversified and specialized (Fleming and John Kress 2011), potentially facilitating reciprocal adaptation.

An overarching hypothesis driving our research is that a shared evolutionary history between *Piper* and *Carollia* has led to suites of traits in both groups that represent adaptations to fruit–frugivore interactions. Furthermore, we hypothesize that competition among *Piper* species and among *Carollia* should lead to differential specialization and adaptation among co‐occurring species within both genera. Here, we test several predictions derived from these hypotheses: (1) patterns of interactions between co‐occurring *Piper* and *Carollia* should reflect niche differentiation with certain *Piper* species being consumed more heavily by certain *Carollia*, and (2) differential consumption of *Piper* among *Carollia* should be associated with: (a) bat morphological traits that influence foraging and feeding, and (b) fruit morphological traits that influence frugivore attraction and seed dispersal. To evaluate support for these predictions, we conducted a detailed survey of dietary composition in *Carollia* and developed a modeling framework to simultaneously measure the role of traits of both bats and fruits in structuring their ecological interactions. We use Bayesian generalized joint attribute modeling (GJAM) to estimate the consumption indices—an indication of relative consumption rates—of three co‐occurring species of *Carollia* for *Piper* species, as well as the influence of bat traits on these estimates. In turn, we relate *Piper* fruit traits to these estimates, testing their influence on fruit consumption by bats. Analyzing the trophic interactions among bats and plants, and among competing congeners, requires the integration of several types of ecological data (*e.g.*, continuous traits, presence/absence of food resources), and has been historically challenging to model (Clark 2016; Clark et al. 2017). Joint attribute modeling is able to account for multiple sources of variation and multiple predictors of different data types to obtain robust estimates of responses (Clark et al. 2017).

## 2 Materials and Methods

To evaluate our predictions derived from a hypothesis of reciprocal adaptation between *Piper* and *Carollia*, we collected data from co‐occurring individuals of bats and plants at La Selva Biological Reserve, Sarapiquí, Costa Rica. We used these data to build three types of Bayesian models. The first models link bats and their traits to *Piper* species present in bat feces, generating a set of coefficients that describe how each bat trait or species designation shapes the relative consumption tendency for each *Piper* species. We call these modeled coefficients of bat species and traits “*Piper* consumption indices”. The second model estimates the relationship between bat morphometric (e.g., body size) and performance (*i.e.*, bite force) traits related to feeding, and the third quantifies the effects of *Piper* traits on modeled *Piper* consumption indices by each bat species.

### 2.1 *Piper* Consumption by Bats

To determine how *Carollia* species and traits relate to the consumption of different *Piper* species, we quantified the diets of the three co‐occurring *Carollia* species at La Selva. All procedures for bat capture and handling were approved by the Institutional Animal Care and Use Committee (IACUC) of the University of Washington (UW), Seattle, USA (protocol #4307–02). We used mist nets to capture bats between 1800 and 2200 h along trails throughout the forest during the wet season when there is a greater incidence of fruiting peaks for *Piper* (July and September–December 2015). We collected fecal samples from 318 individuals from the three *Carollia* species (Figure 1): *C. perspicillata* (*N* = 84), *C. sowelli* (*N* = 111), and *C. castanea* (*N* = 123) by placing individual bats in cloth bags for up to 2 h. If the bat defecated, we collected fecal pellets, which we dried in an air‐conditioned room for 1–2 days. Samples were then transported to UW for seed identification. We identified seed species in rehydrated fecal pellets using morphological characters and by comparison to a seed reference library that included *Piper* and non‐*Piper* species native to La Selva. The reference library was built from seeds removed from ripe fruits collected directly from the parent plant, and plants were identified by L.B.M., Z.A.K., S.R.W., and Orlando Vargas (OTS), and confirmed via genetic markers (see Santana et al. 2021). If we could not identify the species of a particular seed, we classified them as a morphotype (e.g., *Piper* Type 1). We coded each plant species as present or absent in the individual fecal sample (Data S1).

### 2.2 Bat Traits

We selected several traits that may directly relate to bat functional ecology and fruit selection or consumption. Body size, represented by both body mass (g) and forearm length (mm) has numerous biological implications and may affect dispersal distance (larger ranges may mean larger diversity of fruits [Ritchie and Olff 1999; though see Bloch, Stevens, and Willig 2011]), bite force (larger size and thus stronger bite force may mean proclivity for harder fruits [Santana, Dumont, and Davis 2010]), and foraging sites (larger bats forage in primary forest where fruits are larger at lower densities [Fleming 1991]). Life history traits such as age and reproduction may also affect *Carollia*–*Piper* mutualisms. For example, juvenile *Carollia* have shown a behavioral preference for mid‐ or late‐succession *Piper*, indicating a potential preference for denser foraging habitats. In terms of sex and reproduction, lactating *Carollia perspicillata* females were found to have a low‐prevalence of nitrogen rich foods such as *Piper* (Bohlender et al. 2018). We recorded mass, forearm length, age class (adult, sub‐adult, juvenile), sex (male, female), and reproductive condition (reproductive, non‐reproductive) (Data S1) for each bat that produced a fecal sample. Using these bat‐specific variables as covariates, we built a model to estimate *Piper* consumption indices, which describe the relationship between bat traits or bat species designation and the probability that a given *Piper* species will be represented in the feces (*i.e.*, to examine how bat traits and species designation influence their dietary records). Our data set was composed of multiple data types, including a zero‐inflated matrix of *Piper* species in the bat fecal samples (*e.g.*, 0 if *Piper* species is absent; 1 if *Piper* species is present) and correlates of those data: discrete categories of *Carollia* species, continuous bat size traits, as well as the categorical traits of sex and reproductive condition. Simultaneously estimating relationships among bat species, their traits, and the *Piper* species consumed by bats, is a challenge to general linear models. We implemented the flexible framework of generalized joint attribute modeling (GJAM) (Clark et al. 2017), which uses a Bayesian multivariate approach to infer the parameters of the linear model based on a series of joint distributions of both the bat traits and the *Piper* fecal abundances, while simultaneously accommodating multifarious trait data, in this case from bats.

### 2.3 Bat Functional Traits

Bite force is a metric of feeding performance linked to the mechanical demands of the food a species can process (Aguirre et al. 2002; Santana, Dumont, and Davis 2010; Santana 2016; Santana and Miller 2016). Foraging and fruit handling in phyllostomids is known to differ as a function of bite force at the intraspecific level, but differences at shallower evolutionary scales are lesser known (Dumont 1999; Santana and Miller 2016). When bats consume *Piper*, both biting the infructescence and stripping the stalk of *Piper* are important food handling behaviors that vary by *Piper* species (Aguirre et al. 2003). Following methods by Santana, Dumont, and Davis (2010), we measured deep bilateral, voluntary bite forces for 10 wild individuals per *Carollia* species using a piezoelectric force transducer (Kistler 9203; range ± 500 N, accuracy 0·01–0·1 N) attached to a handheld charge amplifier (Kistler 5995A). The force transducer was mounted between two metal plates covered with medical tape to provide a non‐skid biting surface and to protect the bats' teeth. We adjusted the distance between the bite plates for each individual to accommodate a moderate gape angle of approximately 30°, following (Santana, Dumont, and Davis 2010). To avoid variation from age (Santana and Miller 2016) and stress to reproductive females, we only measured adult males and adult non‐pregnant, non–lactating females. We recorded five to eight measurements for each bat and chose the highest value to represent maximum bite force. Following bite force measurements, we recorded head length, width, and height measured to the nearest 0.1 mm (Figure S1B), as well as mass and forearm length for most individuals (Data S1).

### 2.4 *Piper* Fruit and Seed Traits

Physical traits of fruits and seeds can constrain whether and how bats of different sizes can process them. We collected dimensions of whole *Piper* infructescences (the unit consumed by *Carollia*, called “fruits” throughout this paper for simplicity) and individual seeds to estimate how these traits relate to the modeled *Piper* consumption indices. We measured length and width from five ripe fruits from each *Piper* species to the nearest 0.001 mm, and used ImageJ (Rasband, W.S., ImageJ, US National Institutes of Health, Maryland, USA) to measure seed length and seed width from digital photographs of three seeds from each fruit. Seed photos were taken with a Leica MZ 95 microscope camera coupled with Clemex Captiva software. We used these fruit and seed measurements to calculate a ratio (length/width) as an estimate (index) of fruit and seed shape, respectively.

### 2.5 Generalized Joint Attribute Modeling

For each observation i of n bat individuals, there is a set $\;{\left\{x_{i},y_{i}\right\}}^{n}$, in which each $x_{i}$ observation has Q predictors to result in a vector of predictors $x_{\mathrm{iq}}:1\ldots Q$. In our case Q=6, with predictors being bat species, age class, sex, reproductive condition, mass, and forearm length. The set of responses is a vector of $y_{\mathrm{ip}}:1\ldots P$, where P is the total number of *Piper* species (P=18) observed across all fecal samples. For $y_{\mathrm{ip}}$, each vector of bat individual i is the presence or absence of *Piper* species p. Seven *Piper* species were removed from the analysis, as they accounted for less than 1% of the observations (Figure S2). Most of the observations in $y_{\mathrm{ip}}$ are 0, meaning most *Piper* species are not observed in a sample. To accommodate this zero‐inflation, GJAM implements a Tobit regression. The representations of $x_{i}$ and $y_{i}$ are composed of partitions of discrete and continuous space, and GJAM applies a connection between the two, which we represent as I in our model. Thus, it is possible to estimate a continuous response $w_{i}$ from multifarious data such that for each observation,

$$w_{i}\left|x_{i}\right.,y_{i}\sim N\left(B^{\prime },E\right)\times I$$

where $B^{\prime }$ is the matrix of coefficients and E is a P×P correlation matrix to represent the covariances among the response variables. For detailed explanations of the calculations of I, E, and w, see further discussion in Clark et al. (2017). We estimated the coefficients using the R package gjam v. 2.1.6 for 20,000 generations, discarding 4000 as burn‐in. As discussed in Taylor‐Rodríguez et al. (2017), we applied a series of dimension reduction options (*N* = 2, 5, by *r* = 2, 5) to facilitate convergence amidst the multiple dimensions of covariance space and adopted the one that yielded the lowest model deviance. We compared both fractional composition models (continuous on [0, 1]) and presence–absence models (discrete). Medians of the posterior distributions of the continuous response $w_{i}$ were used for further modeling.

### 2.6 Bayesian Hierarchical Modeling

After determining that both sex and head length were linear predictors of bite force in regressions with either a sample‐wide intercept (male sex coefficient *t*_(27)_ = 2.29, *p*‐value = 0.03, head length coefficient *t*_(27)_ = 7.60, *p*‐value = 3.54e‐08), or species‐specific intercepts (male sex coefficient *t*_(27)_ = 4.23, *p*‐value 1.20e‐04, head length coefficient *t*_(27)_ = 2.44, *p*‐value = 0.01), we modeled bite force as a function of bat body size traits while controlling for both sex and head length, which may explain bite force. We used Jags v.3.3.0 (Plummer 2003) to code these models, and ran them in the R package R2jags v.0.04–01 (Su and Yajima 2012). These models included species‐specific intercepts with priors drawn from a normal distribution. Priors for both between‐ and within‐population variances were modeled as half‐Cauchy distributions with a variance of at least 100,000. These priors do not make any assumptions about the relative contribution of variation from different levels in the hierarchy (Gelman and Hill 2006). For each model, four independent chains ran for 500,000 iterations with 250,000 iterations as burn‐in, and samples were taken every 250 generations. Convergence was assessed by both the effective sampling size of model parameters (> 1000 in every case), and the potential scale reduction factor (PSRF), which approaches 1 at convergence (Gelman and Rubin 1992). The models coded measures of error to estimate the variance explained, as outlined by Gelman and Pardoe (2006).

We used the *Piper* traits as regressors in Bayesian models of *Piper* consumption indices by bat species using GJAM. Thus, these models connect the differential use of *Piper* resources by bats (*e.g.*, across species, age class, or body sizes) to the *Piper* traits that might underlie those differences. We used the R package MCMCglmm (Hadfield 2010) to code the models, and accounted for the correlation structure of the data due to evolutionary relatedness by including a molecular phylogeny of *Piper* (Santana et al. 2021) as a species‐specific (random) effect. We applied a parameter‐expanded prior with the parameters *V* = 1 *ν* = 1 for the residual variance (Rojas et al. 2018), and a proper Cauchy prior defined by *V* = 0.5 *ν* = 1 and *αμ* = 0 and *αV* = 10^3^ for the random term (Hadfield 2019). Each model ran for 200,000 iterations, sampling every 100, with 10,000 generations as burn‐in. Convergence of the resulting posteriors was assessed by the effective sampling size of model parameters (> 1000 in every case). In total, we ran four models. Each one had the bat *Piper* consumption indices associated with the following: (1) bat forearm, (2) body mass, (3) *C. castanea* , and (4) *C. perspicillata* , as a response and the *Piper* traits as predictive variables. A statistically supported relationship between a bat *Piper* consumption index and a *Piper* trait would indicate fruit traits are associated with consumption as modeled by GJAM.

## 3 Results

As expected, the percentage of *Piper* presence in the diet was highest in the specialist *C. castanea* (67.5%) and lowest in the generalist *C. perspicillata* (45.2%). *C. sowelli* was intermediate (60.5%) (Figure S2). For the period sampled, *Piper* Type 4 was the most common species in the diets of *C. castanea* and *C. sowelli* , while *P. hispidum* was the most common for *C. perspicillata* . Dietary proportions are displayed in Figure S2 and raw diet data in Data S1.

### 3.1 *Piper* Consumption Indices Across Different *Carollia*

**FIGURE 2:**
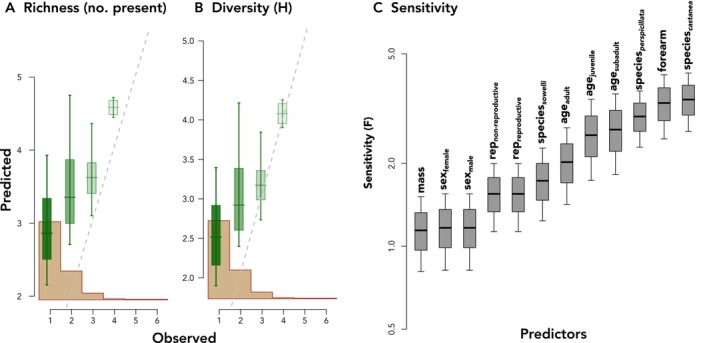
(A) Richness and (B) diversity metrics of posterior predictive checks for the GJAM relative abundance fractional composition model. The brown histogram in (A) and (B) is the frequency distribution of the observed data and the dashed lines are the 1:1 diagonals of the observed values and predictions. (A) Richer and (B) more diverse samples had a better fit. (C) Sensitivity of the model to covariate inputs can be interpreted as the amount of information each input contributes overall to estimating the model coefficients. The higher the sensitivity, the more informative the covariate to the model.

**FIGURE 3:**
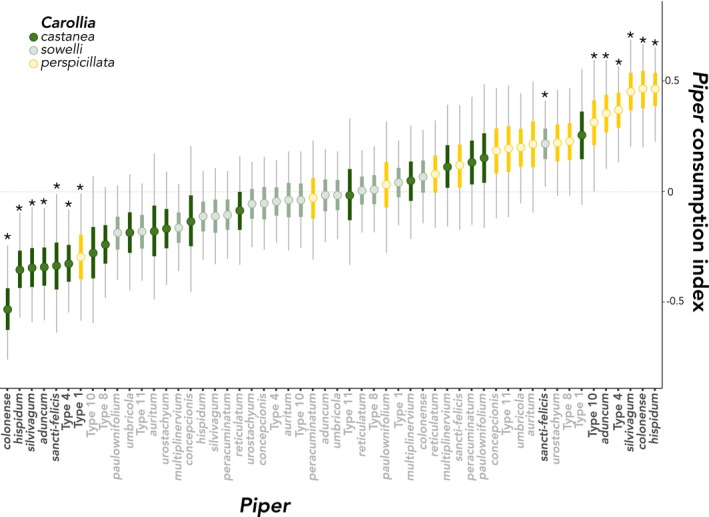
Posterior distributions of model coefficients (*Piper* consumption indices per bat species), ordered by median. *Piper* consumption indices can be interpreted as the probability of a particular *Carollia* species to show a higher or lower consumption index for a particular *Piper* species. Asterisks and black species names refer to *Piper* in which 95% of the highest posterior density intervals did not cross zero, indicating a strong positive or negative response.

**FIGURE 4:**
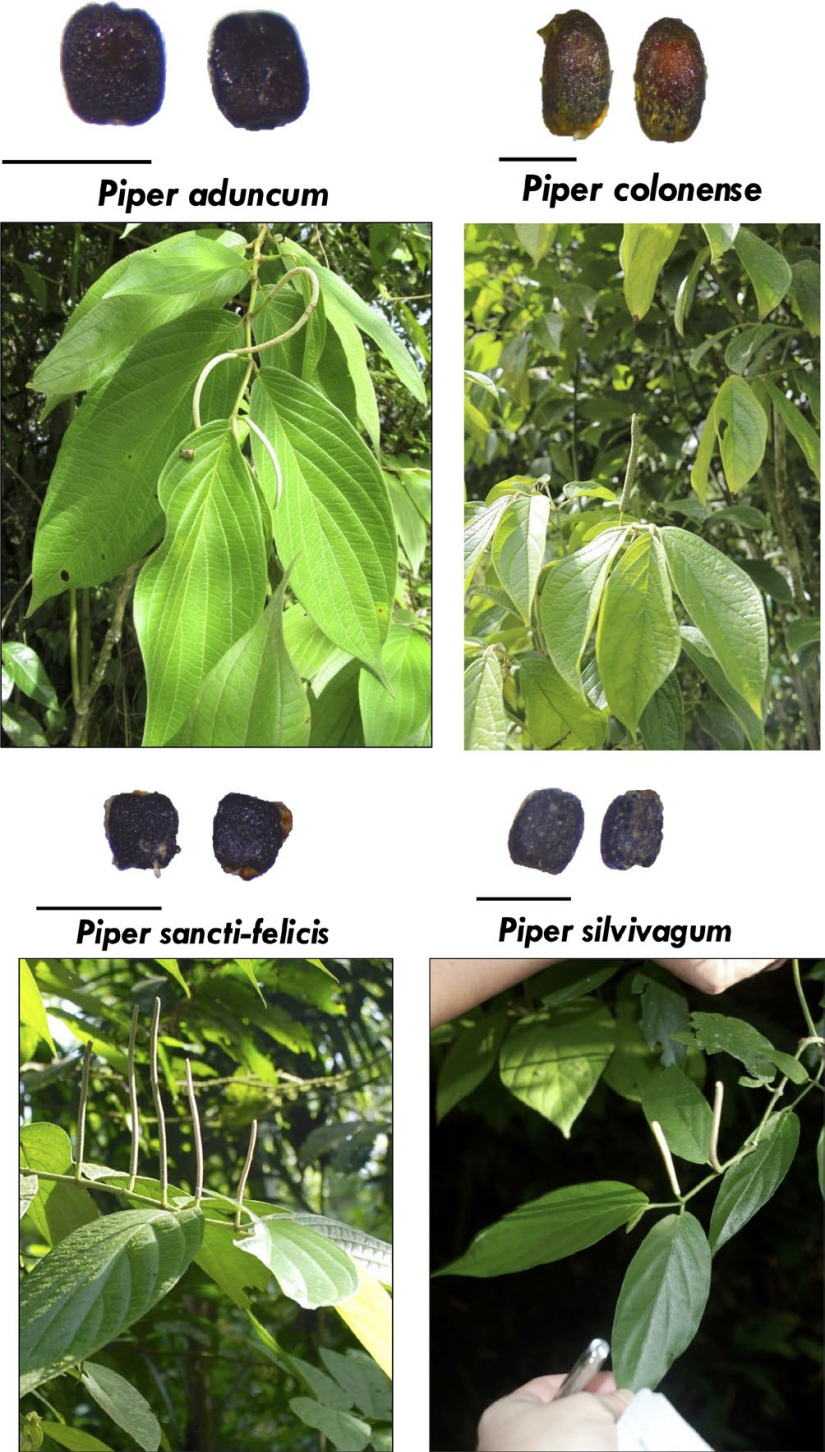
A subset of *Piper* plants, and their seeds after being sampled from bat feces. Scale bar is 1 mm. The *Piper* species shown here are notable outliers of our analyses in Figure 3 and/or Figure 5. Photographs by Sharlene Santana and Leith Leiser‐Miller.

Model fit was assessed through DIC (Deviance Information Criterion) and posterior predictive output. The fractional composition model (as opposed to presence–absence) demonstrated a better fit (Figure 2A,B). In GJAM, the sensitivity of the model to various covariate inputs (*i.e.*, the bat traits and species designations) can be interpreted as the amount of information each input contributes to estimating the model coefficients (Clark et al. 2017). A positive consumption index in a bat species indicates greater relative consumption of that *Piper* species given a particular covariate, while a negative consumption index indicates the opposite. Bat species (particularly *C. castanea* and *C. perspicillata* ), forearm length, age, and weakly reproductive status all showed sensitivity values greater than one, suggesting they were much more informative than sex or body mass in explaining the presence of *Piper* species in the diet of *Carollia* (*i.e.*, *Piper* bat consumption indices; Table S1; Figure 2C). Figure 3 illustrates the *Piper* consumption indices for each bat species, that is, the posterior probabilities for each *Piper* species, estimated by the consumption index of each bat species for that *Piper* species (details in Table S2). While the 95% highest posterior density (HPD) credible interval crossing zero corresponds to a weak relationship between the covariate and the *Piper* species, an HPD not overlapping zero can be interpreted as a strong response. Consequently, six species of *Piper* showed a strong positive response to *C. perspicillata* (in order of highest consumption index: *P. hispidum* (median: 0.45; 95% HPD: [0.22, 0.66]), *P. colonense* (0.45 [0.20, 0.66]), *P. silvivagum* (0.45 [0.20, 0.68]), Type 4 (0.36 [0.13, 0.56]), *P. aduncum* (0.35 [0.10, 0.59]), and Type 10 (0.31 [0.001, 0.59])). The *Piper* specialist *C. castanea* also has the lowest consumption indices for five of these six species (Figure 3): *P. colonense* (−0.52 [−0.75, −0.24]), *P. hispidum* (−0.34 [−0.56, −0.09]), *P. silvivagum* (−0.34 [−0.58, −0.07]), *P. aduncum* (−0.33 [−0.58, −0.07]), and Type 4 (−0.32 [−0.54, −0.08]). *C. castanea* also showed a negative consumption index for *P. sancti‐felicis* (−0.33 [−0.63, −0.01]), toward which *C. sowelli* (the bat species that exhibits intermediate specialization on *Piper*) also demonstrated a positive consumption index (0.22 [0.02, 0.41]). *C. perspicillata* only showed a negative consumption index toward *Piper* Type 1 (−0.29 [−0.58, −0.01]). Table S2 shows coefficient estimates for all *Piper* species. Figure 4 shows a subset of *Piper* species with contrasting outlying responses by bat species.

### 3.2 Influence of Bat Traits on *Piper* Consumption Indices

**FIGURE 5:**
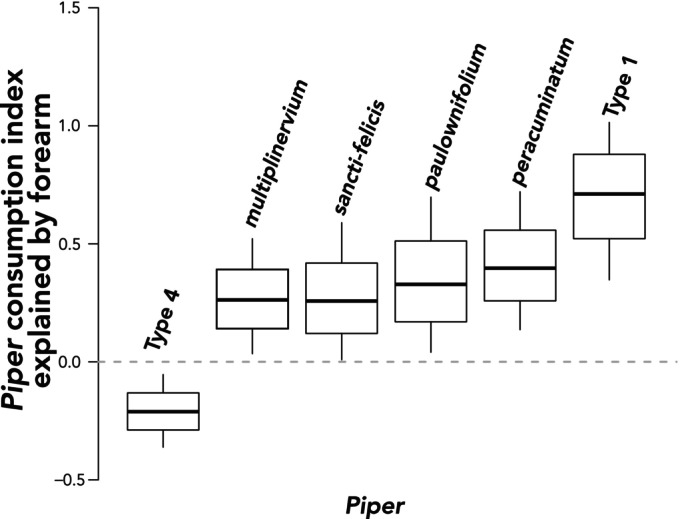
Posterior marginal distributions of model coefficients (*Piper* consumption indices) in response to forearm length, independent of bat speciesand all other considered traits. Coefficients are only shown for *Piper* species that indicate strong positive or negative responses, determined by the entire 95% highest posterior density being entirely above or below zero.

The sensitivity of the model to the bat traits used as model inputs and their influence on consumption indices of *Piper* species varied (Table S1; Figure 2C). The magnitude of these coefficients reflects the influence of the trait on the consumption index, or overall patterns of *Piper* consumption. Sensitivity was high for one covariate representing body size (forearm length), which strongly influenced the consumption indices for several *Piper* species (Figures 2C and 5). The consumption index distribution for *P*. Type 1 showed the strongest positive response to forearm length (0.70 [0.34, 1.01]). There was a strong positive influence of forearm length in four other *Piper* species (Figure 5; *P. peracuminatum*: 0.41 [0.13, 0.73]; *P. paulowniifolium*: 0.34 [0.04, 0.70], *P. sancti‐felicis*: 0.27 [0.01, 0.59], and *P. multiplinervium*: 0.26 [0.03, 0.52]). A strong negative response to forearm was estimated for *Piper* Type 4 (−0.21 [−0.37, −0.06]), which also had anticorrelated consumption indices favoring *perspicillata* (0.36 [0.13, 0.56]) and disfavoring *castanea* (−0.31 [−0.54, −0.08]). Although age showed the second highest sensitivity among all covariates (Figure 2C), no *Piper* species had a posterior that entirely excluded zero (Figure S4), likely because there were few observations of juveniles and subadults. It is worth noting that despite this variation, *P. paulowniifolium* showed the strongest response with adult bats and Type 1 and *P. hispidum* showed the strongest response in juveniles and subadults (Figure S4). There was no meaningful influence of bat sex and very weak influence of reproductive condition on *Piper* species consumed. Table S2 summarizes estimates for each categorical or continuous covariate of this model.

### 3.3 Bat Functional Traits

We modeled the scaling of bite force with head and body dimensions in the natural log scale using hierarchical models in every case. Although head length did not differ among species (*F*_2,27_ = 1.443, *p* = 0.256), it was a positive covariate of maximum bite force with high variance explained (multiple regression *R* ^2^ = 0.90, after controlling for sex), and a consistently positive posterior coefficient distribution (Table S3). Similar results were obtained in combination with body mass (multiple regression *R* ^2^ = 0.90), and forearm length (multiple regression *R* ^2^ = 0.90 and lowest deviance), with the forearm length coefficient indicating negative trends with bite force after controlling for head length (Table S4). Head and forearm length were positively correlated (*R* = 0.76, *t*_28_ = 6.2306, *p* = 9.862e‐07). In short, once the effect of head size is accounted for, and acknowledging that larger bats have larger heads, the marginal relationship between forearm lengths and bite force tends to be negative. Male bats always had greater bite force compared to females, even after controlling for head length or body size (Table S3).

### 3.4 *Piper* Fruit Traits and Bat‐*Piper* Responses

Phylogenetic hierarchical Bayesian models sought to relate *Piper* consumption indices per bat species to *Piper* traits (seed shape index, fruit shape index). These models examined whether *Piper* traits could predict the relative strength of GJAM coefficients reflecting the likelihood that a given bat will consume a given *Piper* species (i.e., *Piper* bat consumption indices). Neither of the fruit traits was a statistically significant predictor of *C. castanea* consumption indices (Table S4), or of consumption indices estimated based on forearm length or body mass for *C. perspicillata* . *C. sowelli* did not have any outlier consumption indices.

## 4 Discussion

Through adaptation, plant–animal interactions may result in the reciprocal influence of fruits on frugivores and vice versa, with suites of matching traits on both sides of the mutualism. However, it is only when ecological traits are measured in both fruits and frugivores simultaneously while considering within‐species variation that one can infer whether measured traits in plant–animal mutualisms are congruent with this scenario. We overcame these challenges by using generalized joint attribute modeling to model the occurrence of *Piper* in bat diets while considering multiple covariates with different variance structures simultaneously. By modeling the influence of both bat and fruit traits on the interaction, we tested whether plant and bat traits predict the structure of bat dietary composition. We discovered bat species identity and functional traits structure the consumption of different *Piper* species, consistent with specialization and niche partitioning. However, the *Piper* traits examined showed no relationship to bat‐*Piper* consumption indices, indicating those plant traits are unlikely to be involved in fruit selection by bats. Despite only subtle trait differences among the bat species studied, our analyses uncovered key differences in consumption potentially contributing to frugivore niche partitioning and therefore adaptation. Our finding that bat species and their traits, primarily body size, drive differential *Piper* fruit consumption supports frugivore specialization.

Consumption of several *Piper* species is non‐random and strongly predicted by the identity of the bat species and forearm length, a strong covariate of body size in bats. Species identity primarily influenced consumption indices, with the co‐occurring *Carollia castanea* and *perspicillata* at opposite ends of consumption index variation. Of the six *Piper* species with the highest consumption index by the generalist *C. perspicillata* , five also had the lowest consumption index by the specialist *C. castanea* (Figure 3). In contrast, previous work found species identity influenced the proportion of *Piper* in fecal samples, but did not affect *Piper* dietary composition by individual bats and, as a result, there was near‐complete dietary niche overlap among the three bat species (Maynard et al. 2019). In those prior analyses, the relationship between species identity and traits was estimated by relating each variable of interest (*e.g.*, traits such as species identity, sex, and age) to distances obtained through non‐metric multidimensional scaling ordination of *Piper* abundance using generalized additive models. Finding an inverse consumption index for a suite of *Piper* species is evidence that *Carollia* bats do partition *Piper* resources, contrary to previous results. Our results suggest GJAM models achieve greater sensitivity by allowing for simultaneous inference of multiple covariates with different variance structures, helping elucidate the patterns of species interaction within this guild, capturing the richness of the samples (Figure 2). While *C. perspicillata* has a more flexible diet that includes many non‐*Piper* fruits (Figure S2) or even nectar and insects, when it eats *Piper*, it uses *Piper* species that *C. castanea* seldom uses. This indicates these bats partition the dietary niche in previously unsuspected ways. In line with previous results, however, nearly all other *Piper* species had overlapping consumption indices for the three bat species (Figure 3), indicating dietary niche overlap among *Carollia* bats for most but not all *Piper* species.

Besides species identity, body size as measured by forearm length also structured consumption indices for some *Piper* species. Consumption indices for *Piper* Type 1 and Type 4 separate *C. castanea* and *C. perspicillata* , and indices for *P. sancti‐felicis* separate *C. castanea* and *C. sowelli* . The consumption indices for these *Piper* species also showed a strong response to bat forearm length, even after accounting for bat species identity. Differences in body size that structure *Piper* consumption indices may indicate differences in dietary niche breadth because niche breadth can increase with body size in bats via larger home ranges (Barclay and Brigham 1991). Instead of specialization, *Piper* consumption indices might be related to *Piper* geographic distribution and bat dispersal ability. In effect, and although we did not focus on non‐*Piper* species, the larger generalist *C. perspicillata* eats a greater proportion of fruits from several other plant genera too. Relating niche breadth to body size would thus support ongoing competition among bat congeners. These two bat species may also use their habitat differently, or at different times, or be in active competition on an ecological time scale. We propose in the presence of a competing species such as *C. perspicillata* , the realized niche is smaller for the specialist *C. castanea* , such that it specializes on different *Piper* resources and reduces niche overlap. In terms of relating to the functional ecology of body size differences, structuring of *Piper* consumption indices by size—which predicts bite force (Table S3)—aligns our results with comparative analyses for all phyllostomids in which bite force relates to consumption of larger and/or tougher fruit (Santana, Dumont, and Davis 2010). While the link between bite force and *Piper* consumption is indirect, our results further support an adaptive hypothesis for bat traits that are structured by fruit consumption.

Under an adaptive scenario and dispersal syndrome hypothesis, a reciprocal association between frugivore phenotype and food resource traits is expected in the context of coevolution in plant–animal mutualisms (Valenta and Nevo 2020), as in the case of beak size and shape and seed size and hardness in Galapagos finches (Schluter and Grant 1984; Schluter, Price, and Grant 1985). We found no relationship between measured *Piper* traits and any consumption indices estimates, suggesting coarse *Piper* fruit morphologies are not adaptions to signal specific *Carollia* frugivores. While there is empirical evidence of fruit morphologies correlating with traits of their dispersers (Janson 1983; Valenta and Nevo 2020), our results suggest morphological traits of the animal disperser likely structure this particular mutualism. Morphological traits of the frugivore appear to be shaped by *Piper* consumption but not the other way around. For plants, slower evolutionary rates in outbreeding populations (Herrera 1984; Valenta and Nevo 2020), and generalism may explain the lack of relationship between measured traits and consumption indices. As with many animal‐dispersed fruiting plants, *Piper* is also consumed by other non‐bat frugivores (*e.g.*, birds) (Palmeirim, Gorchoy, and Stoleson 1989; Sil et al. 2024) for which seed and fruit morphology may play a role, undetected in this study. Our results only represent one site of the *Carollia*‐*Piper* distribution during the wet season; we advocate for future studies to quantify the consumption of *Piper* at different times of year and throughout the sympatric distributions in other parts of Central America.

Unmeasured fruit traits might also be selectively shaped in this plant–bat interaction. Traits such as fruiting time (Thies and Kalko 2004; Sil et al. 2024), plant habitat, or secondary metabolite profiles (Whitehead, Obando Quesada, and Bowers 2016; Santana et al. 2021) have been proposed as more important to differential consumption than the physical traits of fruit we measured. There is also strong support for chemical communication between plants and bats, with behavioral evidence for *Carollia* using the sense of smell to locate ripe fruit (Thies, Kalko, and Schnitzler 1998; Leiser‐Miller et al. 2020), and bat olfactory receptor diversity scaling to dietary diversity (Yohe et al. 2021). Chemical bouquet composition both differs sharply and evolved adaptively among *Piper* species (Santana et al. 2021), so those traits may affect and better reflect reciprocal adaptation to bat consumption. In short, while we found no effect of fruit and seed dimensions on bat consumption, behavioral and chemical evidence suggest scent traits are likely to be more important in structuring niche partitioning across bat species.

Though the inverse relationship of consumption indices for *Piper* may indicate ongoing specialization in food resources in two *Carollia* species, there is some indication that behavioral aspects, such as learning, also contribute to differential resource use. nNo *Piper* species showed a significant association to bat age, but age had high sensitivity and some patterns of contrasting consumption tendencies in adult versus juvenile bats warrant further exploration (Figure S3). A previous study found that adults used a lower percentage of mid‐to‐late successional species than juveniles, partitioning *Piper* by habitat (Maynard et al. 2019). We hypothesize that older, more experienced bats can locate and exploit resources better than younger, naïve bats, perhaps through spatial learning, or familiarity with less conspicuous fruit cues.

Because our model both accounts for several sources of variation and can incorporate many different types of ecological data, we were able to discover food resource partitioning and estimate the influence of various traits on plant–frugivore interactions. A caveat to other models analyzing similar data sets is that ecological data and covariates are often collected at multiple scales and must be analyzed indepedently given the multifarious nature of the covariates. Numbers of observations among groups and traits also vary and combining continuous and discrete data is not straightforward. Generalized linear models and other hierarchical Bayesian modeling make use of non‐linear link functions whereas our approach (GJAM) uses a “censored” approach that allows discrete and composition data to take on a continuous nature and can be more seamlessly integrated in the same framework (Clark et al. 2017). The resulting marginal distributions also enable a straightforward interpretation of the response to a given covariate, independent from all other model inputs. Identifying such patterns provided quantitative evidence of the relationships between differential resource use and frugivore traits. We discovered that, while the use of different fruit resources is related to putatively adaptive differences in body size traits, age may also play an important role in defining the dietary niche of overlapping species. As body size may confer niche breadth and underlies functional traits such as bite force, our findings are consistent with both specialization through adaptation and ongoing competition among bat frugivores. While there was no effect of the plant traits examined on bat consumption, mounting evidence for plant chemical adaptation and specialization in this system suggests plant–bat interactions is not mediated by gross fruit morphology. Thus, this approach enabled us to both uncover the most informative predictors of differential plant use and hint at new mechanisms underlying the evolutionary ecology of fruit–frugivore interactions.

## Author Contributions

**Laurel R. Yohe:** conceptualization (lead), formal analysis (equal), investigation (lead), methodology (lead), project administration (equal), resources (equal), software (lead), visualization (lead), writing – original draft (lead), writing – review and editing (equal). **Leith B. Leiser‐Miller:** data curation (lead), investigation (supporting), project administration (supporting), resources (lead), validation (supporting), writing – review and editing (supporting). **Zofia A. Kaliszewska:** data curation (supporting), investigation (supporting), resources (supporting), writing – review and editing (supporting). **Susan R. Whitehead:** formal analysis (supporting), investigation (supporting), resources (supporting), validation (equal), writing – review and editing (supporting). **Sharlene E. Santana:** conceptualization (equal), data curation (lead), funding acquisition (lead), investigation (supporting), project administration (equal), resources (lead), supervision (lead), writing – review and editing (supporting). **Liliana M. Dávalos:** conceptualization (lead), formal analysis (lead), funding acquisition (lead), investigation (equal), methodology (lead), project administration (equal), software (lead), supervision (lead), validation (lead), visualization (equal), writing – original draft (lead), writing – review and editing (lead).

## Conflicts of Interest

The authors declare no conflicts of interest.

## Supporting information


**Data S1.** Raw data of individual metadata for each bat, including forearm length, body weight, age class, sex, reproductive condition, and the presence/absence code of observing the seed of the respective plant species in the fecal sample.

Data S2.

## Data Availability

All data, scripts, and results have been deposited onto Dryad (DOI: http://dx.doi.org/10.5061/dryad.2v6wwpzwg).
