# Supplementary material for: Frugivore Traits Predict Plant–Frugivore Interactions Using Generalized Joint Attribute Modeling
Source: Ecol Evol. 2025 Jan 16;15(1):e70772. doi: 10.1002/ece3.70772 (PMC11739623; doi:10.1002/ece3.70772)

Supplemental Material for

Frugivore traits predict plant–frugivore interactions using generalized joint attribute modeling

Authors: Laurel R. Yohe[1,2,3,4], Leith B. Miller[5], Zofia A. Kaliszewska[5], Susan R. Whitehead[6], Sharlene E. Santana[5,7], Liliana M. Dávalos[3,8]

Affiliations:

[1]Department of Bioinformatics and Genomics, University of North Carolina Charlotte, Charlotte, NC, 28223, USA

[2]North Carolina Research Campus, Kannapolis, NC, 28081, USA

[3]Department of Ecology & Evolution, Stony Brook University, 650 Life Sciences Building Stony Brook, NY 11794, USA

[4]Department of Earth and Planetary Sciences, Yale University, 210 Whitney Ave. New Haven, CT 06511, USA

[5]Department of Biology, University of Washington, Seattle, WA, 98195, USA

[6]Department of Biological Sciences, Virginia Tech, Blacksburg, VA 24061, USA

[7]Burke Museum of Natural History and Culture, University of Washington, Seattle, WA, 98195, USA

[8]Consortium for Inter-Disciplinary Environmental Research, School of Marine and Atmospheric Sciences, Stony Brook University, 129 Dana Hall, Stony Brook, NY 11794, USA

Table S1. Sensitivity estimates for each covariate response to *Piper* plant in GJAM model, corresponding to Figure 2C.

Variable	Sensitivity	Standard Error
Bat$_{castanea}$	2.93	0.384
Bat$_{sowelli}$	1.65	0.245
Bat$_{perspicillata}$	2.75	0.345
Forearm	3.15	0.443
Mass	1.32	0.191
Sex$_{\mathrm{female}}$	1.06	0.166
Sex$_{\mathrm{male}}$	1.06	0.166
Age$_{\mathrm{adult}}$	1.91	0.305
Age$_{\mathrm{subadult}}$	2.60	0.433
Age$_{\mathrm{juvenile}}$	2.45	0.407
Rep$_{\mathrm{non-repreproductive}}$	1.42	0.197
Rep$_{\mathrm{reproductive}}$	1.42	0.197

Table S2. Coefficients of response variables for each *Piper* plant species. Estimates are mean prediction values for each variable. SD is posterior standard deviation. The percentage columns are the upper and lower boundary of the 95% credible interval of the posterior distribution. Rows highlighted in **bold** are estimates in which the entire 95% credible interval does not cross zero, the +/− indicating the direction of this estimate.

Piper	variable	Estimate	StdErr	2.5%	97.5%
aduncum	**Bat*castanea***	**−0.333**	**0.128**	**−0.575**	**−0.072**
	**Bat*perspicillata***	**0.348**	**0.124**	**0.102**	**0.589**
	Bat*sowelli*	−0.015	0.106	−0.224	0.191
	Sex_{male}	0.062	0.074	−0.083	0.207
	$Age_{juvenile}$	−0.165	0.197	−0.553	0.215
	$Age_{subadult}$	0.143	0.202	−0.250	0.542
	$Rep_{reproductive}$	0.022	0.142	−0.255	0.299
	mass	−0.045	0.088	−0.218	0.129
	forearm	0.055	0.114	−0.155	0.294
auritum	Bat*castanea*	−0.172	0.167	−0.482	0.172
	Bat*perspicillata*	0.210	0.150	−0.092	0.493
	Bat*sowelli*	−0.038	0.113	−0.262	0.180
	Sex_{male}	0.016	0.078	−0.137	0.168
	$Age_{juvenile}$	−0.066	0.198	−0.454	0.322
	$Age_{subadult}$	0.000	0.210	−0.412	0.419
	$Rep_{reproductive}$	0.066	0.143	−0.212	0.348
	mass	−0.083	0.091	−0.262	0.091
	forearm	0.249	0.172	−0.059	0.606
colonense	**Bat*castanea***	**−0.520**	**0.133**	**−0.750**	**−0.241**
	**Bat*perspicillata***	**0.452**	**0.120**	**0.199**	**0.660**
	Bat*sowelli*	0.067	0.107	−0.141	0.277
	Sex_{male}	−0.080	0.073	−0.225	0.063
	$Age_{juvenile}$	−0.098	0.201	−0.499	0.293
	$Age_{subadult}$	0.015	0.215	−0.407	0.436
	$Rep_{reproductive}$	0.082	0.146	−0.204	0.367
	mass	0.008	0.086	−0.160	0.177
	forearm	−0.052	0.103	−0.240	0.162
concepcionis	Bat*castanea*	−0.129	0.167	−0.448	0.204
	Bat*perspicillata*	0.181	0.149	−0.119	0.464
	Bat*sowelli*	−0.052	0.107	−0.259	0.157

	Sex_{male}	0.065	0.078	−0.086	0.218
	$\text{Age}_{\text{juvenile}}$	−0.096	0.200	−0.492	0.292
	$\text{Age}_{\text{subadult}}$	−0.022	0.214	−0.442	0.392
	$\text{Rep}_{\text{reproductive}}$	0.117	0.146	−0.169	0.408
	mass	−0.160	0.088	−0.334	0.011
	forearm	0.225	0.171	−0.073	0.597
hispidum	**$\text{Bat}_{castanea}$**	**−0.344**	**0.121**	**−0.562**	**−0.092**
	**$\text{Bat}_{perspicillata}$**	**0.453**	**0.109**	**0.224**	**0.646**
	$\text{Bat}_{sowelli}$	−0.110	0.101	−0.304	0.092
	Sex_{male}	0.077	0.068	−0.057	0.210
	$\text{Age}_{\text{juvenile}}$	−0.151	0.174	−0.497	0.188
	$\text{Age}_{\text{subadult}}$	0.278	0.185	−0.092	0.634
	$\text{Rep}_{\text{reproductive}}$	−0.127	0.125	−0.369	0.118
	mass	−0.058	0.080	−0.213	0.098
	forearm	0.097	0.097	−0.085	0.298
multiplinervium	$\text{Bat}_{castanea}$	0.114	0.140	−0.155	0.389
	$\text{Bat}_{perspicillata}$	0.047	0.130	−0.211	0.296
	$\text{Bat}_{sowelli}$	−0.161	0.099	−0.355	0.035
	Sex_{male}	0.088	0.070	−0.050	0.226
	$\text{Age}_{\text{juvenile}}$	0.027	0.181	−0.336	0.380
	$\text{Age}_{\text{subadult}}$	0.025	0.197	−0.356	0.417
	$\text{Rep}_{\text{reproductive}}$	−0.052	0.132	−0.306	0.207
	mass	−0.063	0.080	−0.218	0.093
	**forearm**	**0.264**	**0.125**	**0.032**	**0.523**
paulownifolium	$\text{Bat}_{castanea}$	0.154	0.164	−0.162	0.480
	$\text{Bat}_{perspicillata}$	0.030	0.150	−0.273	0.314
	$\text{Bat}_{sowelli}$	−0.184	0.108	−0.395	0.028
	Sex_{male}	0.042	0.074	−0.104	0.187
	$\text{Age}_{\text{juvenile}}$	−0.069	0.187	−0.440	0.296
	$\text{Age}_{\text{subadult}}$	−0.096	0.205	−0.499	0.306
	$\text{Rep}_{\text{reproductive}}$	0.165	0.139	−0.106	0.442
	**mass**	**−0.240**	**0.087**	**−0.413**	**−0.073**
	**forearm**	**0.340**	**0.172**	**0.038**	**0.700**
peracuminatum	$\text{Bat}_{castanea}$	0.133	0.146	−0.147	0.424
	$\text{Bat}_{perspicillata}$	−0.030	0.135	−0.303	0.229
	$\text{Bat}_{sowelli}$	−0.103	0.100	−0.300	0.093
	Sex_{male}	0.066	0.069	−0.070	0.201

	$Age_{juvenile}$	0.137	0.175	−0.203	0.478
	$Age_{subadult}$	−0.227	0.197	−0.612	0.156
	$Rep_{reproductive}$	0.089	0.128	−0.160	0.341
	**mass**	**−0.213**	**0.079**	**−0.368**	**−0.059**
	**forearm**	**0.405**	**0.150**	**0.134**	**0.725**
reticulatum	$Bat_{castanea}$	−0.084	0.124	−0.327	0.161
	$Bat_{perspicillata}$	0.079	0.123	−0.162	0.319
	$Bat_{sowelli}$	0.005	0.093	−0.182	0.186
	Sex_{male}	0.009	0.067	−0.122	0.142
	$Age_{juvenile}$	0.106	0.177	−0.249	0.445
	$Age_{subadult}$	−0.035	0.195	−0.413	0.350
	$Rep_{reproductive}$	−0.071	0.128	−0.320	0.182
	mass	0.091	0.075	−0.057	0.241
	forearm	−0.010	0.088	−0.180	0.166
sancti-felicis	**$Bat_{castanea}$**	**−0.329**	**0.157**	**−0.630**	**−0.010**
	$Bat_{perspicillata}$	0.114	0.142	−0.170	0.388
	**$Bat_{sowelli}$**	**0.215**	**0.099**	**0.022**	**0.408**
	Sex_{male}	0.018	0.070	−0.118	0.157
	$Age_{juvenile}$	−0.192	0.184	−0.554	0.172
	$Age_{subadult}$	0.145	0.193	−0.237	0.516
	$Rep_{reproductive}$	0.046	0.131	−0.212	0.305
	mass	−0.017	0.080	−0.175	0.138
	**forearm**	**0.266**	**0.150**	**0.006**	**0.589**
silvivagum	**$Bat_{castanea}$**	**−0.336**	**0.130**	**−0.582**	**−0.068**
	**$Bat_{perspicillata}$**	**0.446**	**0.123**	**0.200**	**0.680**
	$Bat_{sowelli}$	−0.110	0.110	−0.324	0.106
	Sex_{male}	0.057	0.076	−0.091	0.205
	$Age_{juvenile}$	−0.101	0.198	−0.484	0.285
	$Age_{subadult}$	0.014	0.211	−0.405	0.424
	$Rep_{reproductive}$	0.087	0.147	−0.196	0.378
	mass	−0.082	0.089	−0.256	0.093
	forearm	−0.068	0.097	−0.249	0.133
umbricola	**$Bat_{castanea}$**	**−0.182**	**0.133**	**−0.442**	**0.079**
	**$Bat_{perspicillata}$**	**0.197**	**0.125**	**−0.051**	**0.441**
	$Bat_{sowelli}$	−0.015	0.100	−0.213	0.181
	Sex_{male}	0.121	0.072	−0.019	0.263
	$Age_{juvenile}$	0.108	0.173	−0.231	0.449

	$Age_{subadult}$	0.019	0.189	−0.356	0.386
	$Rep_{reproductive}$	−0.127	0.125	−0.373	0.120
	mass	−0.133	0.080	−0.293	0.025
	forearm	0.030	0.101	−0.161	0.236
urostachyum	$Bat_{castanea}$	−0.164	0.130	−0.417	0.090
	$Bat_{perspicillata}$	0.219	0.122	−0.019	0.456
	$Bat_{sowelli}$	−0.055	0.098	−0.247	0.134
	Sex_{male}	0.040	0.070	−0.096	0.177
	$Age_{juvenile}$	−0.024	0.177	−0.370	0.323
	$Age_{subadult}$	−0.009	0.195	−0.381	0.376
	$Rep_{reproductive}$	0.032	0.132	−0.222	0.292
	mass	−0.061	0.080	−0.217	0.094
	forearm	0.123	0.097	−0.067	0.313
Type 1	$Bat_{castanea}$	0.251	0.156	−0.058	0.549
	**$Bat_{perspicillata}$**	**−0.292**	**0.145**	**−0.576**	**−0.007**
	$Bat_{sowelli}$	0.041	0.097	−0.150	0.229
	Sex_{male}	−0.072	0.070	−0.208	0.067
	$Age_{juvenile}$	0.175	0.180	−0.172	0.529
	$Age_{subadult}$	0.034	0.199	−0.361	0.416
	$Rep_{reproductive}$	−0.210	0.131	−0.462	0.048
	mass	0.081	0.079	−0.076	0.234
	**forearm**	**0.699**	**0.176**	**0.341**	**1.016**
Type 4	**$Bat_{castanea}$**	**−0.318**	**0.119**	**−0.540**	**−0.080**
	**$Bat_{perspicillata}$**	**0.361**	**0.112**	**0.132**	**0.561**
	$Bat_{sowelli}$	−0.043	0.094	−0.228	0.142
	Sex_{male}	−0.015	0.066	−0.145	0.114
	$Age_{juvenile}$	−0.103	0.176	−0.445	0.239
	$Age_{subadult}$	0.017	0.193	−0.357	0.400
	$Rep_{reproductive}$	0.085	0.127	−0.160	0.338
	mass	−0.013	0.075	−0.162	0.135
	**forearm**	**−0.213**	**0.079**	**−0.366**	**−0.056**
Type 8	$Bat_{castanea}$	−0.234	0.126	−0.474	0.021
	$Bat_{perspicillata}$	0.225	0.122	−0.017	0.463
	$Bat_{sowelli}$	0.010	0.098	−0.181	0.203
	Sex_{male}	0.040	0.070	−0.099	0.173
	$Age_{juvenile}$	−0.006	0.184	−0.371	0.353
	$Age_{subadult}$	0.025	0.200	−0.366	0.419

	$Rep_{reproductive}$	−0.019	0.133	−0.282	0.239
	mass	−0.026	0.080	−0.180	0.131
	forearm	0.102	0.098	−0.089	0.295
Type 10	$Bat_{castanea}$	−0.269	0.169	−0.587	0.069
	**$Bat_{perspicillata}$**	**0.307**	**0.149**	**0.001**	**0.590**
	$Bat_{sowelli}$	−0.037	0.110	−0.251	0.180
	Sex_{male}	−0.035	0.077	−0.185	0.116
	$Age_{juvenile}$	0.048	0.193	−0.334	0.421
	$Age_{subadult}$	−0.129	0.210	−0.537	0.284
	$Rep_{reproductive}$	0.081	0.142	−0.199	0.359
	**mass**	**−0.184**	**0.090**	**−0.364**	**−0.009**
	forearm	0.231	0.172	−0.068	0.602
Type 11	$Bat_{castanea}$	−0.011	0.168	−0.326	0.328
	$Bat_{perspicillata}$	0.190	0.150	−0.113	0.475
	$Bat_{sowelli}$	−0.179	0.111	−0.398	0.037
	Sex_{male}	0.110	0.079	−0.043	0.263
	$Age_{juvenile}$	−0.063	0.199	−0.453	0.330
	$Age_{subadult}$	0.016	0.211	−0.402	0.434
	$Rep_{reproductive}$	0.047	0.144	−0.237	0.332
	mass	−0.069	0.091	−0.250	0.107
	forearm	0.291	0.180	−0.028	0.667

Table S3. Results from hierarchical maximum likelihood regressions of bite force as a function of bat individual and species traits. Each covariate corresponds to a mean measurement per bat species *i* in species *k*. Deviance is a measure of how well the model fits the data, with higher values of deviance indicating the data deviates substantially from model predictions. Estimates in bold a 95% posterior distribution that does not cross zero.

Formula	Estimate	Mean	2.50%	97.50%
$bite.force_i \sim \alpha_k + \beta_1 * head.length_i + \beta_2 * body.mass_i + \beta_3 * male.sex_i + \varepsilon_i + \varepsilon_k$				
	$\boldsymbol{\alpha_{castanea}}$	**−4.686**	**−9.345**	**−0.273**
	$\alpha_{perspicillata}$	−3.933	−8.828	0.639
	$\alpha_{sowelli}$	−3.899	−8.737	0.625
	$\boldsymbol{\beta_1}$	**1.932**	**0.434**	**3.517**
	β_2	−0.074	−0.51	0.341
	$\boldsymbol{\beta_3}$	**0.412**	**0.194**	**0.623**
	ε_i	0.162	0.118	0.229
	ε_k	2.623	0.259	16.003
	Deviance	−21.808	−28.401	−9.991
$bite.force_i \sim \alpha_k + \beta_1 * head.length_i + \beta_2 * forearm.length_i + \beta_3 * male.sex_i + \varepsilon_i + \varepsilon_k$				
	$\alpha_{castanea}$	3.151	−5.756	11.161
	$\alpha_{perspicillata}$	4.165	−5.165	12.597
	$\alpha_{sowelli}$	4.024	−5.195	12.307
	$\boldsymbol{\beta_1}$	**1.470**	**0.281**	**2.718**
	β_2	−1.772	−4.15	0.669
	$\boldsymbol{\beta_3}$	**0.270**	**0.140**	**0.403**
	ε_i	0.155	0.117	0.21
	ε_k	2.971	0.306	18.582
	Deviance	−28.504	−34.851	−17.332
$bite.force_i \sim \alpha_k + \beta_1 * head.length_i + \beta_3 * male.sex_i + \varepsilon_i + \varepsilon_k$				
	$\alpha_{castanea}$	−2.34	−5.828	1.251
	$\alpha_{perspicillata}$	−1.599	−5.266	2.199
	$\alpha_{sowelli}$	−1.63	−5.26	2.074
	$\boldsymbol{\beta_1}$	**1.176**	**0.005**	**2.328**
	$\boldsymbol{\beta_3}$	**0.261**	**0.127**	**0.401**
	ε_i	0.16	0.12	0.215
	ε_k	3.258	0.249	19.544

Deviance	−26.424	−31.925	−16.462

Table S4. Results from phylogenetic Bayesian regressions of GJAM *Piper* consumption indices for *C. castanea* as a function of *Piper* species traits. Each covariate corresponds to a mean measurement per *Piper* species *i*. $\text{Piper}^{\text{Cca}}$ represents the proclivity of *castanea* for each *Piper* species estimated by the GJAM model. Each covariate corresponds to a mean by *Piper* species *i*. α, intercept, β coefficient, Σ phylogenetic variance, ε_i residual variance.

formula	parameter	mean	2.5%	97.5%
$\text{Piper}^{\text{Cca}}{}_i \sim \alpha + \beta *$ seed $\text{ratio}_i + \Sigma + \varepsilon_i$	intercept (α)	−0.083	−0.724	0.719
	β	−0.052	−0.572	0.457
	Σ	0.039	0.000	0.160
	ε	0.160	0. 051	0.317
$\text{Piper}^{\text{Cca}}{}_i \sim \alpha + \beta *$ infructescence.$\text{ratio}_i + \Sigma + \varepsilon_i$	intercept (α)	−0.045	−0.491	0.458
	β	−0.016	−0.016	0.017
	Σ	0.028	0.000	0.122
	ε	0.131	0.038	0.268

Figure S1. Illustration of A) lateral view of *Carollia castanea* head cranium and dorsal view showing linear measurements of head dimensions taken from live bats. HL is head length, HH is head height, HW is head width and B) set up for measuring bite force from bats *C. castanea.* Photo credit: David Villalobos Chavez.

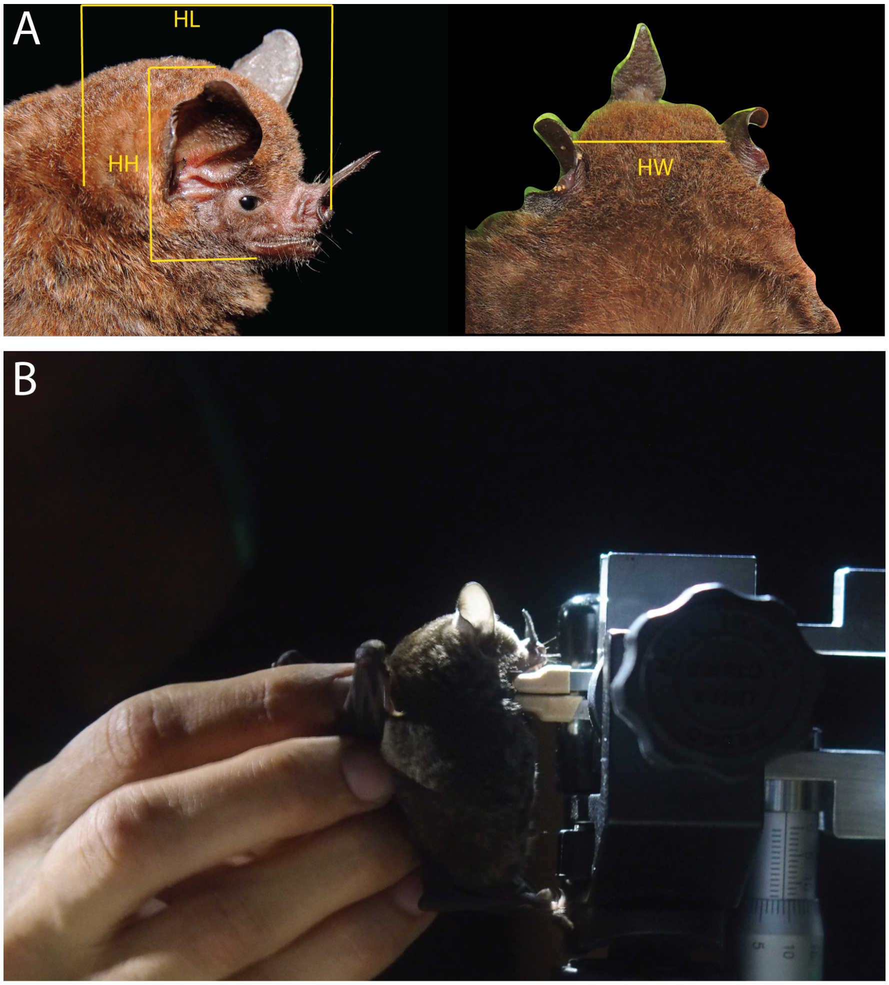

Figure S2. Percentage of non–*Piper* and *Piper* species in the diet of three *Carollia* bats. Note that Type 7, *P. auritifolium*, *P. biolleyi*, *P. decurrens*, *P. peltatum*, *P. terrabanum*, and *P. trigonum* have been removed as they account for less than 1% of the diet. See Data S1 for full spreadsheet.

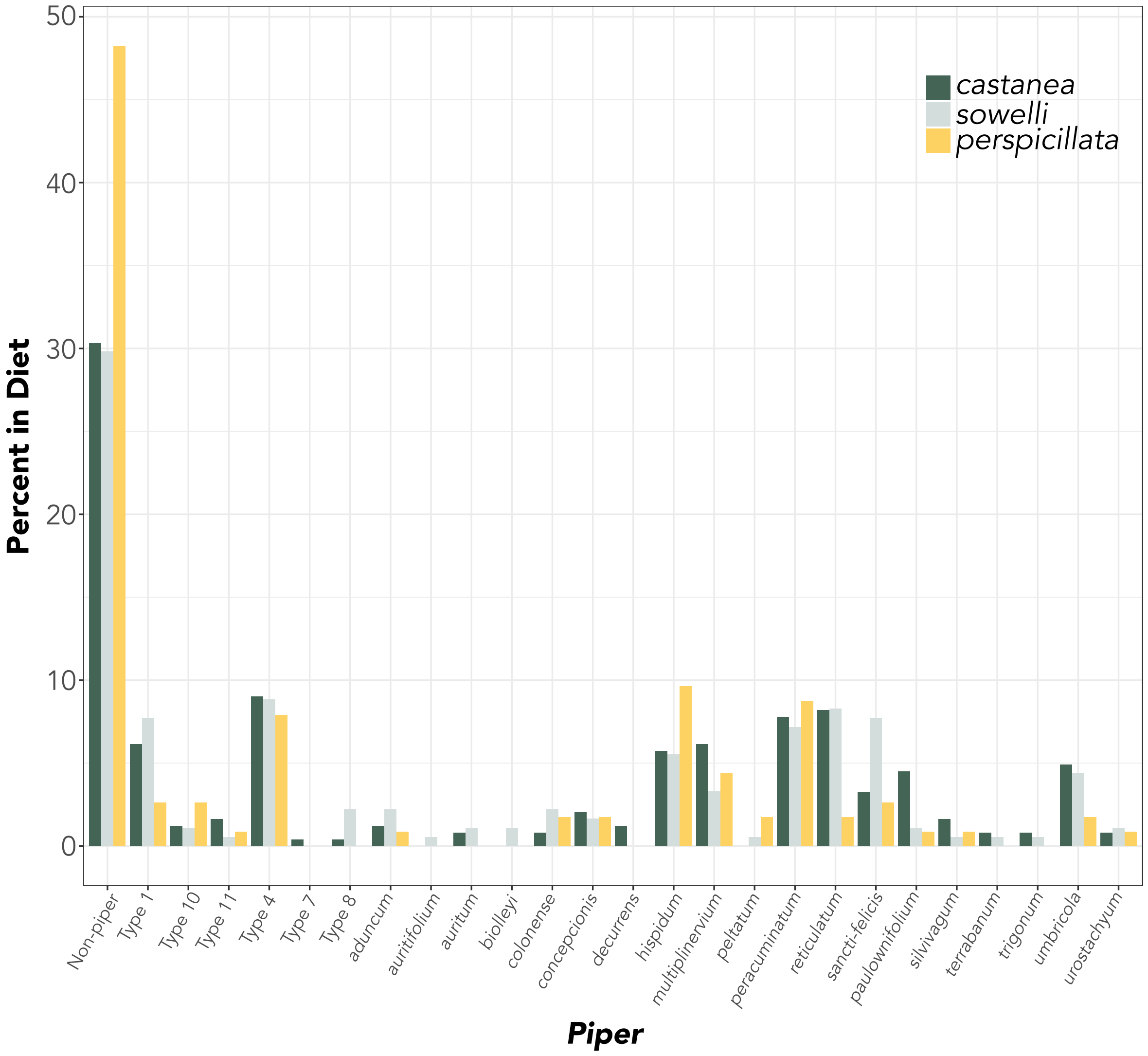

Figure S3. Body size measurements for three bat species, showing the variation in body mass (A) and forearm length (B). Forearm length is a standard and often diagnostic measure of size for bats.

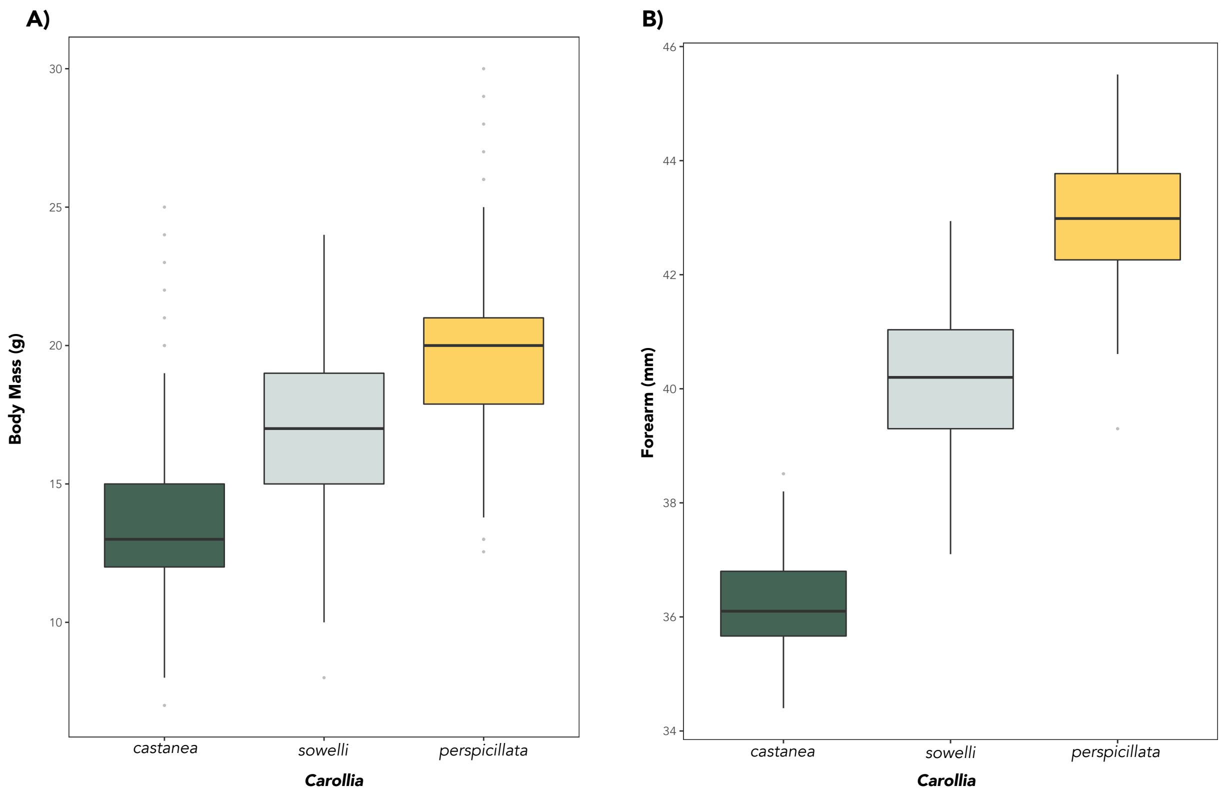

Figure S4. Posterior distributions of age responses for each *Piper* species, ordered by median. Probabilities represent the influence of age category on *Piper* consumption index.

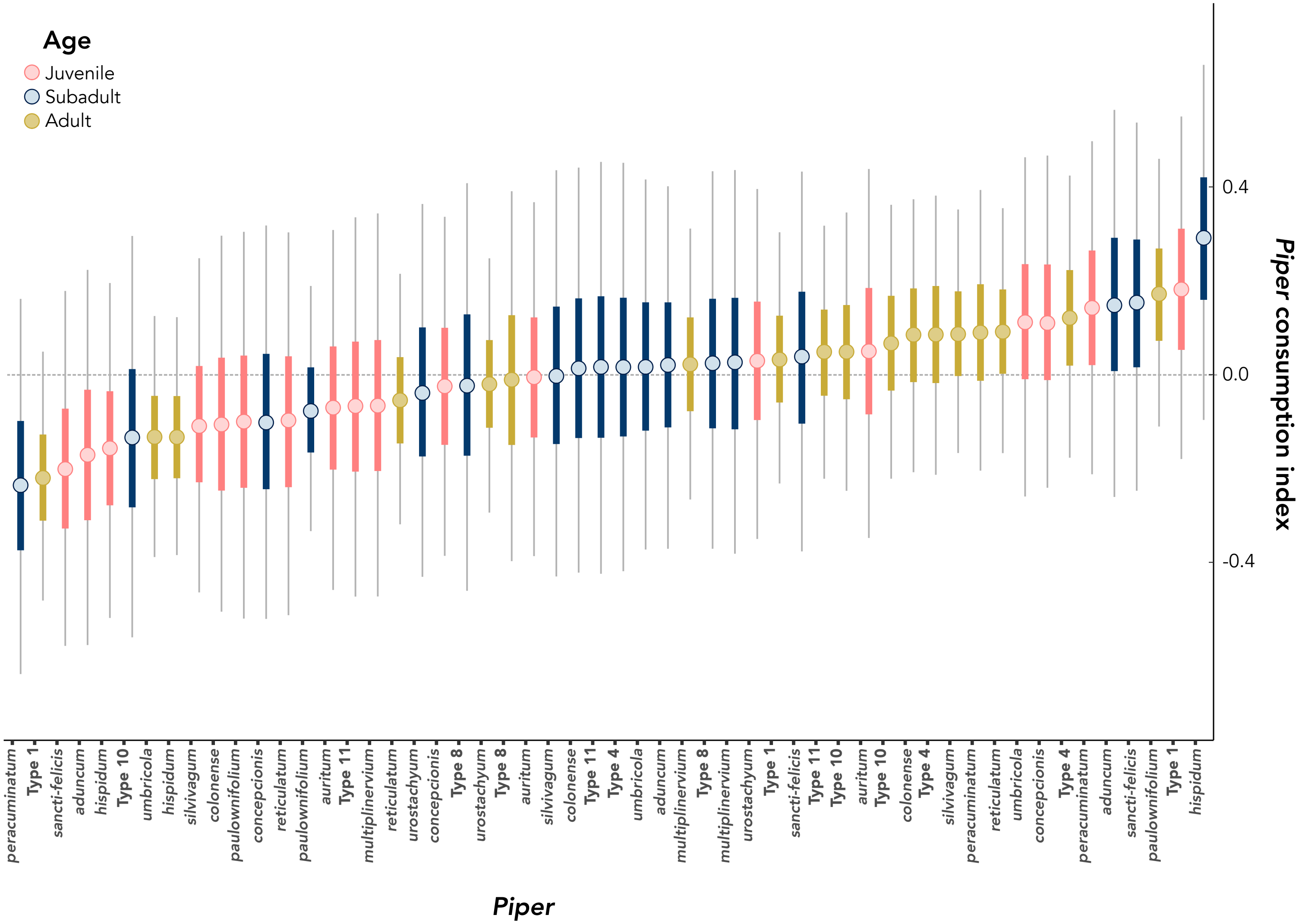

Supplement: Supplementary file 2 — Data S2. [file ECE3-15-e70772-s001.pdf]
